# Regional Differences in mRNA and lncRNA Expression Profiles in Non-Failing Human Atria and Ventricles

**DOI:** 10.1038/s41598-018-32154-2

**Published:** 2018-09-17

**Authors:** Eric K. Johnson, Scot J. Matkovich, Jeanne M. Nerbonne

**Affiliations:** 1 Department of Medicine, Cardiovascular Division, Washington University School of Medicine, St. Louis, MO 63110 USA; 2 Department of Developmental Biology, Washington University School of Medicine, St. Louis, MO 63110 USA

## Abstract

The four chambers of the human heart play distinct roles in the maintenance of normal cardiac function, and are differentially affected by inherited/acquired cardiovascular disease. To probe the molecular determinants of these functional differences, we examined mRNA and lncRNA expression profiles in the left (LA) and right (RA) atria, the left (LV) and right (RV) ventricles, and the interventricular septum (IVS) of non-failing human hearts (N = 8). Analysis of paired atrial and ventricular samples (n = 40) identified 5,747 mRNAs and 2,794 lncRNAs that were differentially (>1.5 fold; FDR < 0.05) expressed. The largest differences were observed in comparisons between the atrial (RA/LA) and ventricular (RV/LV/IVS) samples. In every case (e.g., LA vs LV, LA vs RV, etc.), >2,300 mRNAs and >1,200 lncRNAs, corresponding to 17–28% of the total transcripts, were differentially expressed. Heterogeneities in mRNA/lncRNA expression profiles in the LA and RA, as well as in the LV, RV and IVS, were also revealed, although the numbers of differentially expressed transcripts were substantially smaller. Gender differences in mRNA and lncRNA expression profiles were also evident in non-failing human atria and ventricles. Gene ontology classification of differentially expressed gene sets revealed chamber-specific enrichment of numerous signaling pathways.

## Introduction

The four chambers of the human heart play distinct roles in the generation, coordination and maintenance of normal cardiac rhythms, and are differentially affected in inherited and acquired cardiovascular disease(s). The molecular determinants of regional differences in the structural and functional properties of the human heart, as well as the impact of cardiac or systemic disease on these mechanisms, however, are not well understood. Although transcriptional profiling of human heart has been reported^1–6^, most of the previous studies have used unpaired tissue samples, obtained from multiple different hearts^2,4,5^ and have been limited to the analysis of mRNA expression in a small number, typically two, of different regions^1,2,5,6^. Human tissue samples collected for transcript or other analyses are inherently heterogeneous, being derived from individuals of different ages, sexes, and other demographics, as well as distinct health and medical histories. The potential negative impact of this heterogeneity, however, can be minimized by the use of paired (matched) tissue samples from individual hearts. Indeed, the use of paired tissue samples for differential expression analysis has been shown to increase statistical power, compared with non-paired samples^7^. Paired tissue samples from different regions (left and right ventricles, left and right atria and interventricular septum) of individual donor hearts, therefore, were used in the studies completed and presented here.

Transcription of mammalian genomes is complex, and includes the expression of many different RNA species^8^. A large portion of the genome, for example, is transcribed as long noncoding RNAs (lncRNAs), a heterogeneous group of noncoding transcripts longer than 200 nucleotides, residing within or between (also called long intergenic RNA or lincRNA) coding regions^9^. LncRNAs have been shown to be functional in a variety of physiological and pathophysiological processes through epigenetic, transcriptional and post-transcriptional mechanisms^9^. Although probably best known as regulators of transcription, including epigenetic modification of chromatin, lncRNAs have also been shown to regulate post-transcriptional processes, including mRNA translation, pre-mRNA splicing, mRNA decay, and the expression/function of miRNAs and transcription factors^9^. Indeed, lncRNAs appear to be multifunctional and, in addition, their functions are not dependent solely on sequence (like miRNAs) or structure (like RNA-binding proteins). Rather, lncRNAs function is determined by *both* sequence *and* structure, forming molecular frameworks and scaffolds for the assembly of macromolecular regulatory complexes^9^. In the heart, *Braveheart*, a cardiac-specific lncRNA, has been shown to be required for epigenetic regulation of cardiac lineage commitment in mouse embryonic stem cells^10^. Several studies have also linked lncRNAs to the physiological regulation of cardiac function^11^ and to pathophysiological cardiac hypertrophy and remodeling^11–15^. In spite of this, lncRNAs have not been previously included in analyses of regional differences in transcriptome profiles in the human heart.

Here, we used RNA Sequencing (RNASeq) to provide global transcriptome profiling of cardiac mRNAs and lncRNAs from transmural tissue samples obtained from the left (LA) and right (RA) atrial appendages, left (LV) and right (RV) ventricles, and the interventricular septum (IVS) of non-failing human hearts (N = 8). Differential expression analysis of paired samples (n = 40) identified regional differences in both the mRNA and lncRNA expression profiles in the atria and ventricles, as well as in comparisons between the LA and RA, and the LV, RV and IVS. Principal component and differential expression analyses of mRNA and lncRNA expression profiles also revealed marked male-female differences. Functional classification of differentially expressed transcripts revealed chamber-specific differences in numerous signaling pathways.

## Methods

All studies were performed in accordance with relevant guidelines and regulations, and were approved by the Washington University Institutional Review Board. The high-throughput sequencing data obtained in this study have been deposited at the National Center for Biotechnology Information Gene Expression Omnibus https://www.ncbi.nlm.nih.gov/geo/query/acc.cgi?acc=GSE112339, and can be accessed with input of the following code: ensxesmslryrfqt.

### Tissue acquisition and RNA isolation

Non-failing human hearts (N = 8), declined for transplantation, were obtained from Mid-America Transplant Services (MTS) in St. Louis, MO. MTS is a non-for profit organization which provides human organs and tissues for human transplantation. Tissues not used for transplantation are made available for research pursuant to the informed consent received by MTS from the donor’s legal representative; no tissues were obtained from prisoners. The demographics of the donors are summarized in Supplemental Table 1. Transmural tissue samples from the left (LV) and right (RV) ventricles, left (LA) and right (RA) atrial appendages and interventricular septum (IVS) of each heart were collected in the operating room at MTS, immediately flash-frozen in liquid nitrogen and stored at −80 °C. Total RNA was isolated from individual tissue samples using previously described methods^16^. Briefly, total RNA was extracted from ~50 mg of tissue using Trizol reagent with a polytron homogenizer; individual samples from each heart were processed simultaneously. Following RNA isolation, samples were DNAse treated using the RNeasy Mini Kit (Qiagen). RNA quality was assessed using an Agilent 2100 Bioanalyzer. The RNA Integrity Number (RIN) was ≥7.1 for all samples; mean ± SEM RIN was 8.7 ± 0.1, (n = 40).

### Library preparation, sequencing, and data processing

Indexed RNA libraries were prepared from 1 ug of RNA from each sample with the TruSeq RNA Sample Preparation v2 Kit (Illumina). Briefly, RNA was purified by poly-A selection using Oligo(dT)-attached magnetic beads. Purified RNA was eluted, fragmented and reverse transcribed into first strand cDNA, followed by second-strand cDNA synthesis. The resulting double-stranded cDNA was end-repaired, the 3′ end was adenylated, and Illumina sequencing adapters were added to the 3′ and 5′ ends. cDNA libraries were PCR-amplified in the presence of indexing adapters and pooled for RNA-sequencing. To minimize batch effects, library preparations for chamber samples from each individual heart were performed simultaneously. Paired-end 2 × 101 sequencing was performed at the Genome Technology Access Center (GTAC) at Washington University School of Medicine (St. Louis MO) on an Illumina HiSeq. 2500 sequencer. Samples were multiplexed and all five regions of each heart were pooled and run on a single lane to minimize lane effects. After demultiplexing sequencing data, individual libraries were converted to the FASTQ format. Sequence reads were aligned with Tophat^17^ (v2.1.1) to the human genome (Ensembl hg19 release for mRNAs, Ensembl hg38 release for lncRNAs). To quantitate sequence reads, htseq-count from the HTSeq^18^ package was used in union mode together with the standard Ensembl gtf annotation (mRNAs together with a small number of lncRNAs) or a combined gtf comprising the standard Ensembl annotation together with a comprehensive set of lncRNAs from NONCODE2016^19^. For quantification of relative transcript expression levels, mRNAs and lncRNAs read counts were calculated separately.

### Analysis of sequencing data

For differential expression analysis, only transcripts with ≥1 count per million (CPM) reads, in at least 6 (of the 8) samples of each region (i.e. LV vs. LA), were included. Differential expression analysis of paired samples was performed in R-studio using the raw read count values for each gene and pairwise comparisons with the EdgeR software package using the ‘edgeR-robust’ modification and a generalized linear model^20–22^. Libraries were normalized across samples using the trimmed mean of the M-values (TMM). Transcripts was considered differentially expressed if the Benjamini-Hochberg adjusted p-value (FDR)^20^ was <0.05 with a fold change ≥1.5. Gene Ontology (GO) analysis using the differentially expressed gene sets for individual comparisons were performed using the Protein Analysis Through Evolutionary Relationships (PANTHER) Classification System to identify enriched GO Biological Process (BP) and Cellular Component (CC) terms^23,24^. Kyoto Encyclopedia of Genes and Genomes (KEGG) pathway analyses of differential gene sets were performed using the Database for Annotation, Visualization and Integrated Discovery tool (v6.8)^25,26^. Enriched KEGG pathways were considered significant if *P* values were <0.05. Principal component analyses were performed using the Multiple Experiment Viewer (MeV, mev.tm4.org)^27^. Unsupervised hierarchical clustering was performed using Pearson’s correlation with Partek Genomic Suite version 6.6. Spearman’s rank-order correlation analysis was performed by constructing correlation matrices in R with the rcorr function from the Hmisc package^28^.

### Quantitative real-time PCR

For quantitative real-time PCR (RT-qPCR) analyses, total RNA was isolated from 8 male and 8 female LA and RA appendage samples as described above; 2 addition male and 6 additional female LA and RA samples were analyzed along with the 6 male and 2 female samples used for RNA sequencing. Single strand cDNA was produced from 2 µg of total RNA using the High Capacity cDNA kit (Applied Biosystems). Expression levels of *XIST, EIF1AY, TRPC1, GABRA5, SPN, and DDN*, transcripts were determined with SYBR Green (Applied Biosystems) using a 7900HT Fast Real-Time PCR System (Applied Biosystems); primers are provided in Supplemental Table 2. Data were analyzed using the threshold cycle relative method with TATA-Box Binding Protein (*TBP*) as the endogenous control.

## Results

### RNASeq Gene Alignment

A total of 40 indexed RNA libraries were generated from tissues (n = 40) dissected from non-failing human hearts (N = 8; 47 ± 4 years); available demographic information on the donors is provided in Supplemental Table 1. From each (N = 8) heart, five paired, transmural tissue samples were obtained; these were from the left (LV) and right (RV) ventricles, the left (LA) and right (RA) atrial appendages, and the interventricular septum (IVS). The mean ± SEM (n = 40) number of read pairs generated by RNASeq from each sample was 25,726,331 ± 1,514,831. In each sample, more than 76% (21,850,864 ± 1,341,173, range 76.6 to 88.0%, mean = 83.2 ± 0.6%) of the read pairs aligned to the human genome, and more than 93% (20,697,966 ± 1,270,973, range 93.9 to 95.4%, mean = 94.7 ± 0.1%) of the aligned read pairs were uniquely mapped (Supplemental Table 3).

The vast (85.8 ± 0.3%) majority of the uniquely mapped read pairs corresponded to mRNA transcripts, with lncRNAs accounting for the remainder (14.2 ± 0.3%) (Supplemental Fig. 1A). Similar numbers of mRNA transcripts, with at least one count per million reads (1 CPM), were identified for each sample, representing ~54% (12,887 ± 58) of all genes in the human genome as defined by the standard Ensembl annotation (Supplemental Table 3). The majority (73.4 ± 0.4%) of the mRNA reads were identified as nuclear transcripts, and the remainder (26.6 ± 0.4%) mapped to the mitochondrial genome (Supplemental Fig. 1B). Similar numbers of lncRNAs, expressed at ≥1 CPM, were also identified in each sample, corresponding to ~19% (27,656 ± 819) of all lncRNAs defined in NONCODE2016^19^ (Supplemental Table 3). For each region, only the mRNA and lncRNA transcripts, expressed at ≥1 CPM in at least 6 (of 8) samples were considered expressed and analyzed further.

### mRNA expression signatures distinguish non-failing human atria and ventricles

**Figure 1:**
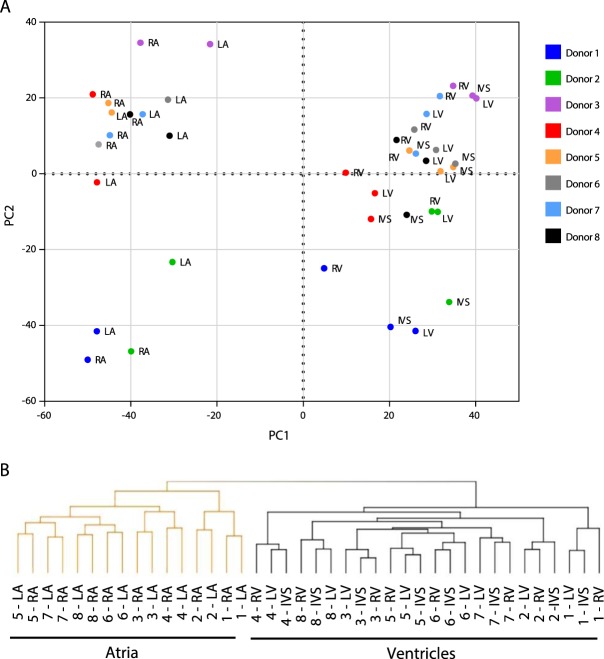
mRNA expression profiles in non-failing human atria and ventricles are distinct. (**A**) Principal component analyses of normalized read counts in all cardiac expressed mRNA transcripts distinguish the atrial and ventricular samples. The left (LA) and right (RA) atrial samples and, separately, the left (LV) and right (RV) ventricular and interventricular septum (IVS) samples cluster by donor. (**B**) Unsupervised hierarchical clustering of the expression profiles of human cardiac mRNAs also distinguish the atrial (orange), from the ventricular (black), samples. These analyses further clustered the LA and RA samples, as well as the LV, RV and IVS samples, by donor.

Principal component analysis using the normalized read counts from all cardiac expressed mRNAs, without incorporating any data on which donor heart was used for isolation of particular samples, clearly discriminate the atrial, from the ventricular, samples (Fig. 1A). Principal component analysis did not discriminate LA from RA samples, or the individual ventricular (LV, RV and IVS) samples. Rather, the LA and RA samples, and the LV, RV, and IVS samples were grouped together by donor (Fig. 1A); the samples from donor 1 and 2 were separated from the other donors in the second principal component (PC2). The groupings of the LA/RA and the LV/RV/IVS samples by donor indicates greater heterogeneity across human hearts, compared with samples from the same heart. Importantly, this observation reveals an important strength of the experimental design strategy, and, in addition, led to the decision to perform subsequent between-chamber differential expression analyses in a paired manner that relate samples to the donor heart of origin. Unsupervised hierarchical cluster analysis of the cardiac mRNA expression profiles also revealed distinct expression signatures in the atria and ventricles (Fig. 1B). The expression profiles for the LA and RA samples, as well as for the LV, RV, and IVS samples, were also grouped by donor using unsupervised hierarchical clustering (Fig. 1B).

To examine regional (e.g., LA vs LV) differences in mRNA transcript expression profiles, differential expression analyses of paired tissue samples was performed as described in Methods and Materials. Only transcripts with an absolute fold difference ≥1.5fold (FDR < 0.05) in expression levels were considered differentially expressed. A total of 5,747 mRNAs, corresponding to ~44% of all cardiac-expressed mRNAs, were identified as differentially expressed between at least two of the five chamber regions (Table 1). Of note, differences in mRNA expression levels were evident in all chamber comparisons, including comparisons between the atria and ventricles, as well as between the left and right sides of the heart (Table 1). The largest differences in relative mRNA expression were evident in comparisons of the atrial and ventricular regions, with 17.3–26.3% of all expressed mRNAs being differentially expressed (Table 1). The mRNA expression profiles in the LA and RA were quite similar, with relatively few (~6%) differentially expressed transcripts (Table 1). In the ventricular (LV, RV, and IVS) regions, the relative mRNA expression levels were also quite similar, and regional differences in transcript expression were observed for only 0.6–4.4% of the cardiac mRNAs (Table 1).

**Table 1:** Differential Expression of Transcripts in Non-Failing Human Hearts^1^.

	Total	Differentially Expressed	% Differentially Expressed
**mRNAs**			
Total	14,725	5,747	39.0%
**Ventricles vs Atria**			
LA vs LV	13,290	2,675	20.1%
RA vs LV	13,325	3,498	26.3%
LA vs RV	13,379	2,309	17.3%
RA vs RV	13,377	3,088	23.1%
IVS vs LA	13,248	2,624	19.8%
IVS vs RA	13,203	3,357	25.4%
**Atria**			
RA vs LA	13,544	856	6.3%
**Ventricles**			
RV vs LV	13,009	210	1.6%
IVS vs LV	12,876	82	0.6%
IVS vs RV	12,993	435	3.3%
**lncRNAs**			
Total	8,785	2,794	31.8%
**Ventricles vs Atria**			
LA vs LV	5,926	1,303	22.0%
RA vs LV	5,623	1,559	27.7%
LA vs RV	6,354	1,251	19.7%
RA vs RV	5,945	1,470	24.7%
IVS vs LA	6,397	1,331	20.8%
IVS vs RA	5,945	1,493	25.1%
**Atria**			
RA vs LA	6,720	406	6.0%
**Ventricles**			
RV vs LV	5,138	77	1.5%
IVS vs LV	5,547	14	0.3%
IVS vs RV	5,483	82	1.5%

^1^The total number of mRNA and lncRNA transcripts that were expressed at ≥1 CPM in at least 6 (of 8) samples from the same region are provided. The numbers and relative percentages of transcripts with ≥1.5 fold difference in expression (FDR < 0.05) are also provided.

**Figure 2:**
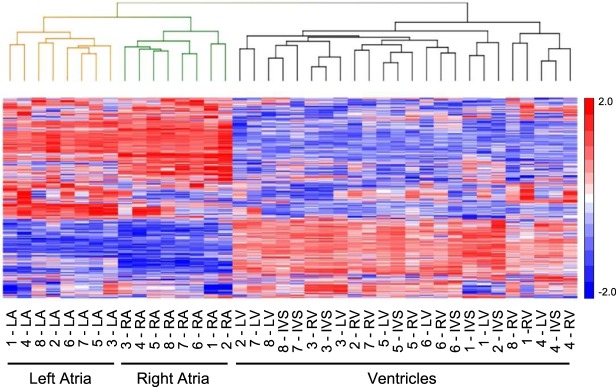
Differentially expressed mRNAs distinguish non-failing human LA and RA. Unsupervised hierarchical clustering and heat map of the expression profiles of the top 100 (greatest difference) differentially expressed cardiac mRNAs distinguish the LA (orange) from the RA (green) samples, as well as from the LV, RV and IVS (black) samples. Normalized read counts were standardized so that the row mean was zero and the standard deviation was 1. Data ranges for higher (red) and lower (blue) expressed mRNAs are indicated.

Unsupervised hierarchical clustering was performed on the top 100 mRNA transcripts differentially expressed in each region to assess whether these transcripts could be considered a “signature” capable of distinguishing regions. As expected, unsupervised hierarchical clustering of the top 100 differentially expressed mRNA transcripts clearly distinguished atrial and ventricular samples (Fig. 2). In addition, hierarchal clustering of the top 100 differentially expressed mRNAs also discriminated the LA from the RA samples (Fig. 2). The LV, RV and IVS samples, in contrast, were not clustered by region, suggesting greater similarity in gene expression in the ventricular (than the atrial) regions (Fig. 2, see Discussion).

### Human atrial and ventricular mRNAs identify chamber specializations

Substantial regional differences in mRNA transcript expression were identified between the atrial and ventricular samples (Table 1). 2,792 mRNAs were expressed at significantly (FDR < 0.05) higher levels in the atria, whereas 2,058 mRNAs showed significantly (FDR < 0.05) higher expression in the ventricles. Complete lists of differentially expressed mRNAs are provided in the data supplement. Among the differentially expressed mRNAs identified, several have previously been reported to be expressed at significantly higher levels in human atria (*NPPA, SLN*, and *MYBPHL*)^29–31^ or ventricles (*MYL2* and *MYL3*)^2,32,33^.

**Figure 3:**
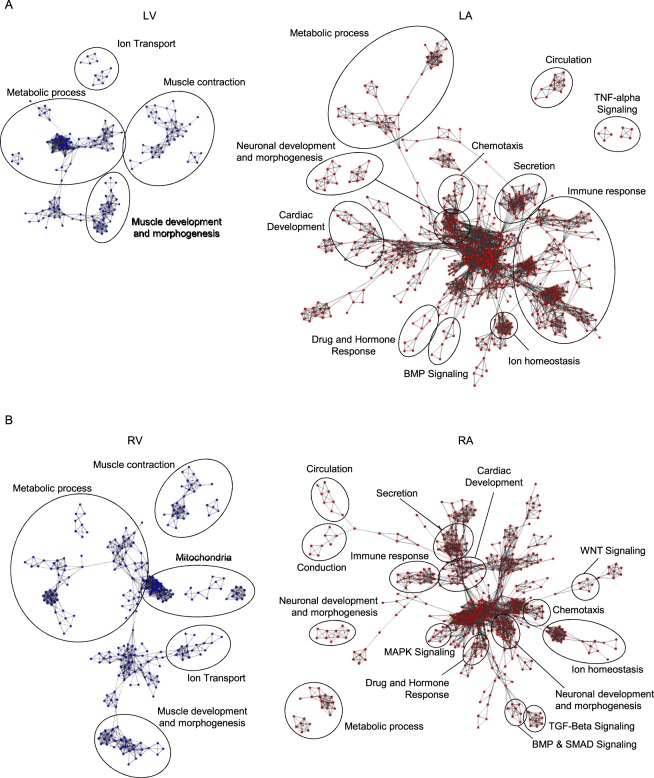
Biological processes enriched in non-failing human atria and ventricles are distinct. Maps of biological processes significantly (*P* < 0.05) enriched in LA vs LV (**A**) and RA vs RV (**B**) were generated using the Enrichment map tool^34^, as described in Methods and Materials. Nodes represent individual biological process terms. Clusters of similarly enriched biological process terms are indicated by the circles; complete lists of circled biological terms, for comparisons between the LA and LV or the RA and RV, are provided in the data supplement.

To determine if the mRNAs differentially expressed in non-failing human atria and ventricles could be classified into distinct functional groups, differentially expressed gene sets were analyzed using Gene Ontology (GO)^24^, as described in Materials and Methods. GO analyses revealed several enriched biological process (BP) terms when comparisons were made between the LA and LV, or between the RA and RV, including 398 and 279 enriched BP terms in the LA and RA, and 110 and 193 enriched BP terms in the LV and RV, respectively. The enriched BPs were assembled into functionally related groups using the Enrichment Map (http://baderlab.org/Software/EnrichmentMap) visualization^34^ tool to visually cluster similar BP terms (Fig. 3). Analyses of the networks identified in the LV, compared with the LA, samples revealed distinct clusters of BP terms associated with metabolism, muscle development, muscle contraction, and ion transport (Fig. 3A, blue). Similar BP clusters were evident in the RV in comparison to the RA (Fig. 3B, blue). Analysis of the networks for the LA compared with the LV, revealed substantially more clusters of BP terms, including clusters associated with immune response, chemotaxis, neuronal development and morphogenesis, ion homeostasis, TNF-alpha signaling and BMP signaling (Fig. 3A, red). Similar complexity of BP terms were also evident when the RA was compared to the RV, and also included clusters of BP terms associated with immune responses, chemotaxis, neuronal development and morphogenesis, and ion homeostasis (Fig. 3B, red). In addition, RA specific clusters of BP terms associated with conduction, MAPK signaling, TGF-beta signaling, WNT signaling, and SMAD signaling were identified (Fig. 3B, see Discussion).

To determine if differentially expressed mRNAs also have distinct subcellular localization patterns, GO analysis, using the Cellular Component classification, was performed (Supplemental Fig. 2). Clusters of enriched cellular components terms in the LV and RV corresponded to the sarcomeres and mitochondria, whereas in the LA and RA, clusters of enriched cellular components terms were associated with membranes, vesicles, synapses, and extracellular space (Supplemental Fig. 2).

### Differences in signaling pathways between the left and right sides of the human heart

The expression levels of the vast majority (>90%) of the mRNAs identified in the LA and RA were similar. Nevertheless, 856 mRNAs were expressed at significantly (FDR < 0.05) different levels in the LA or the RA (Table 1). The mRNAs identified as differentially expressed included transcripts previously reported to have higher expression levels in the human LA (*PITX2*) or RA (*HAMP*, *BMP10*, and *HCN4*)^5,6,35^. GO analysis of the mRNAs differentially expressed in the LA and RA revealed several similar BP terms, including development and morphogenesis, ion homeostasis and secretion.

Differences in enriched BP terms, corresponding to distinct signaling pathways were also identified in the RA (TGF-Beta and MAPK) or the LA (BMP and SMAD). To determine if the mRNAs differentially expressed in the LA or RA correspond to functionally distinct pathways (i.e. different signaling pathways), pathway analysis was performed using the Kyoto Encyclopedia of Genes and Genomes (KEGG), as described in Methods and Materials. KEGG analysis of the differentially expressed mRNAs revealed distinct differences in signaling pathways between the LA and RA (Table 2). In the RA (Table 2), for example, enriched mRNA transcripts associated with WNT, TGFβ, Rap1, Ras, Ca^2+^, and cAMP signaling pathways. In the LA (Table 2), in contrast, enriched mRNA transcripts associated with PPAR, AMPK, and cGMP-PKG signaling pathways (see Discussion).

**Table 2:** Examples of Differentially Enriched Signaling Pathways in Non-Failing Human Right and Left Atria^1^.

KEGG Pathway	KEGG Accession #	Fold Enrichment	*P* Value
**Enriched in RA**			
TGF-beta signaling	hsa04350	3.2	4.0E-02
Rap1 signaling	hsa04015	2.7	2.6E-03
Wnt signaling	hsa04310	2.6	3.5E-02
Ras signaling	hsa04014	2.5	4.7E-03
Calcium signaling	hsa04020	2.5	1.9E-02
cAMP signaling	hsa04024	2.2	3.4E-02
**Enriched in LA**			
PPAR signaling	hsa03320	5.1	3.4E-04
Gap junction	hsa04540	4.3	4.8E-04
Renin secretion	hsa04924	4.1	6.5E-03
Insulin secretion	hsa04911	3.6	6.9E-03
AMPK signaling	hsa04152	2.8	1.5E-02
Cholinergic synapse	hsa04725	2.7	2.7E-02
Serotonergic synapse	hsa04726	2.7	2.7E-02
Glutamatergic synapse	hsa04724	2.6	3.0E-02
cGMP-PKG signaling	hsa04022	2.3	3.1E-02

^1^Kyoto Encyclopedia of Genes and Genomes (KEGG) pathways differentially represented in the paired RA (N = 8) and LA (N = 8) samples, determined as described in Methods and Materials.

The mRNA expression profiles in the three ventricular (LV, RV and IVS) regions were quite similar, and only 556 mRNAs were identified with significant (FDR < 0.05) differences in expression levels among these (LV, RV, IVS) regions (Table 1). The vast majority of differentially expressed mRNAs, however, were higher expressed in the RV, than the LV or IVS (Supplemental Fig. 3A); 151 (of the 210) differentially expressed mRNAs were higher in the RV, than the LV, and 372 (of the 435) differentially expressed mRNAs were higher in the RV, than the IVS (Table 1). The mRNA expression profiles in the LV and IVS were quite similar, and only 82 mRNAs were found to be differentially expressed; of these, 64 (of 82) showed significantly (FDR < 0.05) higher expression in the LV, and 18 were significantly higher in the IVS (Supplemental Fig. 3A). KEGG pathway analyses of the differentially expressed gene sets revealed regional differences in several metabolic and signaling pathways in the LV, RV and IVS (Supplemental Table 4).

### Regional differences in ion channel expression

The electrical properties of the different chambers of the heart are distinct^36^, and regional differences in ion channel gene expression have been identified^37^. The analyses here revealed significant (fold difference >1.5, FDR < 0.05) differences in the relative expression levels of transcripts encoding several ion channel subunits (Supplemental Tables 5 and 6). The largest differences in relative channel subunits transcript expression were evident in comparisons of the atria and ventricles (Supplemental Table 5). Within the atria, for example, mRNAs encoding potassium (i.e. *KCNA5, KCNJ3, KCNJ5, KCNK1, and KCNK3*), calcium (i.e. *CACNA1D* and *CACNA2D2*) and sodium (*SCN1B*) channel subunits were identified with significantly (FDR < 0.05) higher expression levels in both the LA and RA, compared with the LV and RV, samples (Supplemental Table 5). Several other potassium (i.e. *KCNA4, KCNJ2, KCNJ8*), calcium (*CACNA2D1*) and sodium (i.e. *SCN2B* and *SCN4B*) channel subunits, in contrast, with significantly (FDR < 0.05) higher expression levels in the LV and RV, compared with the LA and RA, were also identified (Supplemental Table 5). Regional differences in the relative expression levels of ion channel subunits were also revealed in comparisons of the LA and RA, including significant (FDR < 0.05) differences in the expression of several potassium (i.e. *KCNA4, KCNJ4, KCNJ5*), calcium *(CACNA1D* and *CACNA2D2)* and sodium (*SCN4B*) channel subunits (Supplemental Table 6). In the ventricular regions, there were fewer differentially expressed channel subunit transcripts; examples of which were *CACNA1E*, with significantly (FDR < 0.05) higher expression in the RV, than in the LV or IVS, and *KCNJ4*, with significantly (FDR < 0.05) higher expression in the LV and IVS, than in the RV (Supplemental Table 6).

### Expression signatures of lncRNAs distinguish non-failing human atria and ventricles

**Figure 4:**
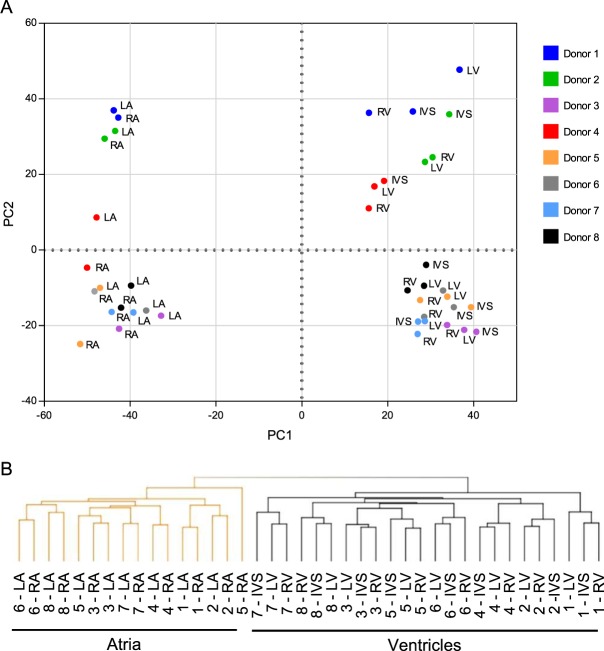
Long non-coding RNAs expression profiles in non-failing human atria and ventricles are distinct. (**A**) Principal component analyses of normalized read counts in all cardiac expressed lncRNA transcripts distinguish the atrial and ventricular samples. The LA and RA samples and, separately, the LV, RV and IVS samples cluster by donor. (**B**) Unsupervised hierarchical clustering of the expression profiles of human cardiac lncRNAs also distinguish the atrial (orange), from the ventricular (black), samples. These analyses further clustered the LA and RA samples, as well as the LV, RV and IVS samples, by donor (see text).

Additional analyses were completed to explore the hypothesis that lncRNA expression profiles might also distinguish the individual regions of the non-failing human heart. Principal component analysis of cardiac expressed lncRNA normalized read counts clearly distinguished the atrial from the ventricular samples (Fig. 4A). In addition, the lncRNA expression profiles of the LA and RA samples, as well as the LV, RV and IVS samples, clustered together by donor (Fig. 4A). Similar to the mRNAs, samples from donor 1 and 2 were separated from the other donors in the second principal component (PC2) (Fig. 4A; see Discussion). Unsupervised hierarchical clustering of the cardiac lncRNAs also revealed distinct expression signatures in the atrial and ventricular samples (Fig. 4B). These analyses further revealed that the expression profiles of the LA and RA samples, as well as the LV, RV, and IVS samples, were also grouped by donor (Fig. 4B).

Differential expression analysis across samples, incorporating the donor heart of origin as a factor, was performed as described for mRNAs. A total of 2,794 lncRNA transcripts, corresponding to ~33% of all cardiac expressed lncRNAs, were identified. Regional differences in the relative expression levels of lncRNAs were identified for all comparisons, including comparisons between the atria and ventricles, as well as between the LA and RA, and between the LV, RV and IVS (Table 1). Complete lists of differentially expressed lncRNAs are provided in the data supplement. The largest differences in relative lncRNA expression profiles were evident in the atria and ventricles, with 19.7–27.7% of the cardiac expressed lncRNAs being differentially expressed (Table 1). The lncRNA expression profiles in the LA and RA were quite similar, with relatively few (6%) differentially expressed transcripts (Table 1). In the individual ventricular (LV, RV and IVS) regions, the relative mRNA expression levels were also quite similar, and regional differences in transcript expression were observed for only 0.3–1.5% of cardiac expressed lncRNAs (Table 1).

**Figure 5:**
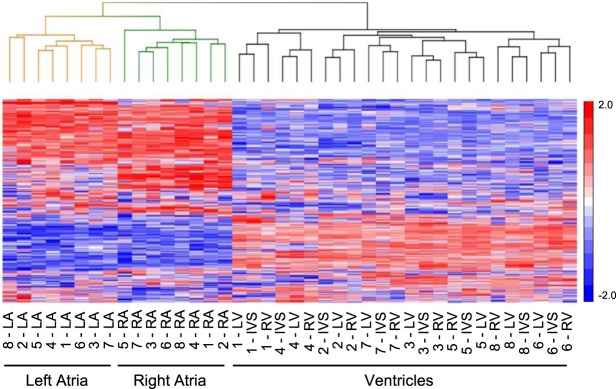
Differentially expressed lncRNAs distinguish non-failing human LA and RA. Unsupervised hierarchical clustering and heat map of the expression profiles of the top 100 (greatest difference) differentially expressed cardiac lncRNAs distinguish the LA (orange) from RA (green) samples, as well as from the LV, RV and IVS (black) samples. Normalized read counts were standardized so that the row mean was zero and the standard deviation was 1. Data ranges for higher (red) and lower (blue) expressed lncRNAs are indicated.

Similar to the mRNA, unsupervised hierarchical clustering of the top 100 differentially expressed lncRNA transcripts clearly distinguished atrial and ventricular samples (Fig. 5). Hierarchal clustering of the top 100 differentially expressed lncRNAs also discriminated the LA from the RA samples (Fig. 5). The LV, RV and IVS samples, instead clustered by donor, suggesting greater similarity in lncRNA expression profiles in the ventricular (than the atrial) regions from individual donors (Fig. 5).

To determine which mRNAs might be correlated with the expression of lncRNAs, Spearman’s correlation analysis^28^ was performed for individual lncRNA and mRNA transcripts for the different regions of the heart, as described in Materials and Methods. A total of 57,487 mRNA-lncRNA *cis* (same chromosome) pairs, with either positive (16,753 pairs, coefficient >0.95) *or* negative (40,734 pairs, coefficient <−0.95) correlations in transcript expression were identified; within each region, 11,497 ± 20 (mean ± SEM) correlations were identified. Of note, correlations in the expression levels of several lncRNAs and ion channel subunit mRNAs were identified, including channel subunits differentially expressed in the atria (*KCNJ3, KCNJ5*, and CACNA1G) or ventricles (*SCN4B* and *CACNA2D1*) (Supplemental Fig. 4).

### Sex differences in gene expression

As noted above, the principal component analyses suggest differences in the mRNA (Fig. 1A) and lncRNA (Fig. 4A) expression profiles in the female (Donors 1 and 2) and male (Donors 3–8) hearts. Several gender differences in the expression of mRNAs and lncRNAs were identified, including transcripts with higher expression in the male (i.e. *EIF1AY* and *DDN*) or the female (i.e. *XIST, SPN, GABRA5* and *TRPC1*) LA and RA samples.

**Figure 6:**
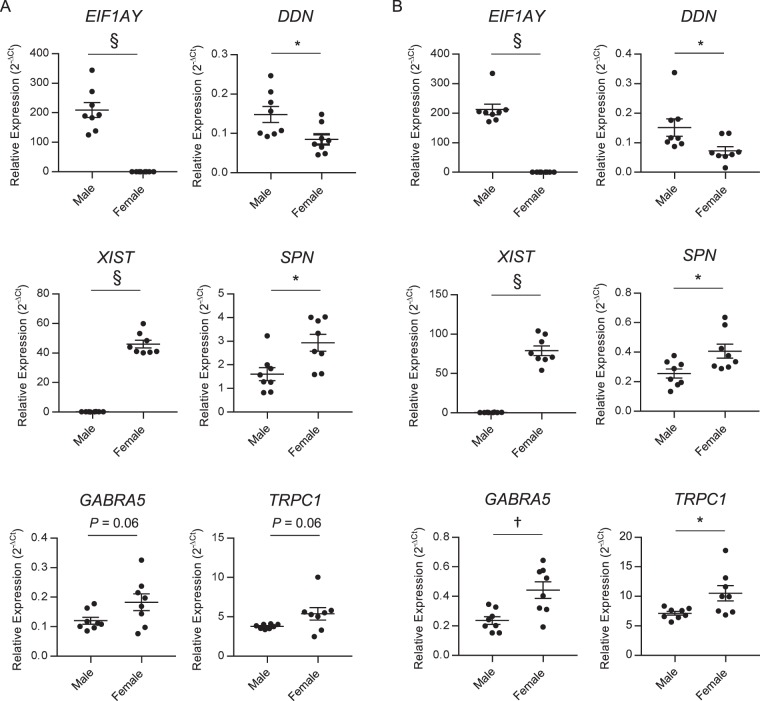
Sex differences in non-failing human heart expression profiles. Comparisons of transcripts encoding genes expressed at higher levels in male (*EIF1AY* and *DDN)* or female (*XIST, SPN, GABRA5* and *TRPC1)* (**A**) LA and (**B**) RA were determined using quantitative RT-PCR (see Methods and Materials). Mean ± SEM (N = 8) values are indicated and the individual normalized expression values are plotted. ^*,†,§^Values indicated are significantly different at the ^*^P < 0.05, ^†^*P* < 0.01 and ^§^*P* < 0.0001 levels.

Due to the limited numbers of male (N = 6) and female (N = 2) samples analyzed by RNA sequencing (see Discussion), the expression levels of several of the differentially expressed transcripts were further investigated in a separate validation cohort of LA and RA samples by RT-qPCR, as described in Methods and Materials. These analyses revealed that the relative expression levels of *EIF1AY* and *DDN* were significantly (*P* < 0.05) higher in male (n = 8) than female (n = 8) LA (Fig. 6A) and RA (Fig. 6B) samples, whereas *XIST* and *SPN* were significantly (*P* < 0.05) higher in female (n = 8) than male (n = 8) LA (Fig. 6A) and RA (Fig. 6B) samples. The relative expression levels of *GABRA5* and *TRPC1* were also significantly (*P* < 0.05) higher in the RA of female (n = 8) than male (n = 8) samples (Fig. 6B). The expression levels of *GABRA5* and *TRPC1* in the female (n = 8) than male (n = 8) samples were trending toward the defined significance cutoff (actual *P* = 0.06) in the LA (Fig. 6A).

## Discussion

Deep RNA sequencing provided comprehensive transcriptional profiling of mRNAs and lncRNAs in the four chambers of the non-failing human heart. Principal component analysis revealed marked differences in mRNA *and* lncRNA expression profiles in comparisons between the atrial and ventricular samples, whereas mRNA and lncRNA expression profiles were similar in the LA and RA, as well as in the LV, RV and IVS. Differential expression analyses of paired tissue samples identified regional differences in the expression levels of numerous mRNAs and lncRNAs. Principal component analysis of the mRNA and lncRNA expression profiles also revealed differences in male and female atria and ventricles, several of which were validated by RT-qPCR.

There is considerable heterogeneity inherent to human samples, and this genetic variability is evident in profound differences in gene expression^38^. An important aspect of the experimental design of this study was the use of paired tissue samples from individual donor hearts for the differential expression analyses. Using paired (matched) tissue samples has been shown previously to increase statistical power significantly compared with non-paired samples^7^. When differential expression analysis was performed here without accounting for the donor origin of the samples, many fewer differentially expressed mRNAs and lncRNAs were identified using the same fold-change and FDR criteria (data not shown). This reduced ability to identify differentially expressed transcripts was particularly evident when comparing cardiac regions with relatively similar expression profiles, such as the LA and RA or the LV, RV and IVS. Indeed, in the absence of pairing, very few or no differentially expressed mRNAs were identified in comparisons of the LV, RV and/or IVS. The benefit of paired samples was further revealed in the principal component analysis and the unsupervised hierarchal clustering, which clearly show that the individual atrial and ventricular samples cluster by donor. These observations are in marked contrast with previous findings obtained in a similarly designed study in the mouse, in which analyses of the expression profiles of transcripts in the LA, RA, LV, RV and IVS samples obtained from multiple mice cluster by region, not by animal^39^. The use of paired tissue samples, therefore, was critical for the identification of regional differences in transcript expression, in particular, between the left and right sides of the heart.

### Regional differences in gene expression

Gene ontology analyses of differentially expressed gene sets in human atria and ventricles revealed that ventricles are enriched in genes associated with muscle contraction and metabolism, whereas atria are enriched in genes associated with signaling and signal transduction. The differentially expressed transcripts identified are consistent with previous studies that examined regional differences in gene expression in human LV and RA^1,2^, as well as in mouse^39^. Several transcripts associated with immune responses were also enriched in the atria, including chemokines (i.e. *CCL2, CXCL1, CXCL14, CXCL16*), interleukins (i.e. *IL1B, IL6, IL8, IL15, IL18*) and interleukin receptors (i.e. *IL1R1, IL4R*). Immunohistochemical analyses have revealed the presence of immune cells, including T-lymphocytes and monocytes/macrophages, in atria from patients in normal sinus rhythm^40^. It will be interesting to determine if the differentially expressed transcripts reflect regional differences in immune cell populations in non-failing human atria and ventricles.

Differences in gene expression were also evident in the LA and RA and, to a lesser extent, the RV, LV and IVS. In the LA and RA, significant differences in expression of several ion channel subunit transcripts were identified. These included channel subunits previously linked to atrial fibrillation, including *SCN3B* and *SCN4B*^41^, which were more highly expressed in the LA, and *KCNJ5* and *HCN4*^41^, which were more highly expressed in the RA. Transcripts of additional atrial fibrillation associated genes, such as those encoding the gap junction proteins *GJA1* and *GJA5*, and the transcription factor, *GATA5*^41^, and signaling pathways were also differentially expressed in the LA and RA. The *WNT* signaling pathway, for example, was enriched in the RA, compared with the LA, and included higher expression of the non-canonical Wnt/Ca^2+^ pathway genes, *WNT5A, FZD2*, *PLCB2* and *PRKCB*.

In addition, comparison of our RNA-sequencing data with a previously published and available non-failing human LV RNA-sequencing data set (ref.^12^, PMID 24429688; https://www.ncbi.nlm.nih.gov/geo/query/acc.cgi?acc=GSE46224) (Supplemental Fig. 5) and to cardiac mRNA data presented in the Medicalgenomics RNAseq Atlas (PMID 22345621 and PMID 20668672; http://medicalgenomics.org/rna_seq_atlas) (Supplemental Fig. 6) revealed very similar transcript expression profiles.

### lncRNAs in human heart

Several lncRNAs have been implicated in cardiac development, functioning, and disease^11^. The cardiac-specific lncRNA, *Myheart* (*Mhrt*), for example, functions to sequester the stress activated factor *Brg1*, a transcriptional regulator that induces aberrant gene expression and cardiac myopathy, and expression of *MHRT* in the human heart is reduced in hypertrophic, ischemic and idiopathic cardiomyopathy^42^. lncRNAs have also been shown to function in regulating ion channel subunit expression. In the mouse, for example, the lncRNA *Kcnq1ot1* can regulate the expression of *Kcnq1*^43^. *KCNQ1OT1* is expressed in humans, and polymorphisms in the *KCNQ1OT1* promoter are associated with prolonged QT intervals^44^. Although limited, transcriptional profiling of lncRNAs in the human heart has also identified several lncRNAs associated with cardiovascular disease(s)^12–15^. The results presented here revealed that several lncRNAs previously associated with cardiovascular disease, such as the Myocardial Infarction Associated Transcript (*MIAT*) and *H19*^11^, are differentially expressed in the atria and ventricles of non-failing human hearts.

The results here identified several lncRNAs with substantial (>10 fold) regional differences in expression in non-failing human hearts. Future studies focused on identifying the functional role(s) of these lncRNAs and the observed differences in lncRNA expression profiles in the non-failing human heart, as well as their possible functional roles in mediating responses to cardiac and systemic diseases, will be of considerable interest.

### Sex differences in gene expression

Principle component analysis revealed clear differences in mRNA and lncRNA expression profiles in the tissue samples from non-failing female and male hearts. Importantly, these differences persisted after X and Y chromosome transcripts were removed from the analysis, demonstrating that the observed gender differences were not due solely to differential expression of genes on the X/Y chromosomes. Due to the fact that the numbers of samples were small, in-depth differential expression analysis of gender differences in the RNASeq transcriptome data was not performed. Subsequent RT-qPCR analysis of a separate donor cohort, however, revealed gender differences in the expression of several transcripts originally identified in the analysis of the RNA-sequencing data.

Numerous previous studies have demonstrated substantial physiological and pathophysiological differences in the hearts of females and males, including gender differences in the prevalence and incidence of heart failure^45^. Electrophysiological differences have also been demonstrated: women, for example, have longer QT intervals and faster resting heart rates, whereas the incidence of atrial fibrillation and sudden cardiac death are higher in men^45^. Women have also been reported to be at higher risk for drug-induced Torsades de pointes when treated with either the antiarrhythmics, sotalol or dofetilide^46^. In contrast, Brugada syndrome, an electrical disorder of the right ventricle, is more prevalent in men than women^47^. The molecular determinants of these differences have not been identified. Studies aimed at elucidating the functional impact of gender differences in mRNA and lncRNA expression profiles, and the molecular mechanisms underlying these differences in the human heart, will be of considerable interest.

## Limitations

Despite the clear advantages of paired tissue samples, the number of donor hearts (N = 8) analyzed in this study was relatively small, and the samples were obtained from a heterogeneous donor cohort (Supplemental Table 1). In addition, although the hearts used were obtained from donors who did not have previous histories of heart failure, different types and levels of tissue/systemic stress, which could affect transcript expression, were likely to be present. As discussed above, regional differences in the mRNA and lncRNA expression profiles by unsupervised hierarchal clustering and principle component analyses were evident in the male and female samples, and several differentially expressed transcripts were validated by RT-qPCR. Nevertheless, the small numbers of samples analyzed precluded unbiased differential expression analysis of the RNASeq data. Additional studies should be conducted on larger numbers of well-annotated clinical samples to generate more complete human cardiac expression databases.

The deep sequencing was performed on RNA isolated from transmural non-failing human heart tissue samples, which are heterogeneous and contain multiple cell types, including cardiomyocytes, fibroblasts, endothelial cells, macrophages, etc.^48^. The acquired transcript data, therefore, reflects mRNAs and lncRNAs contributed by all of these cell types. Recent single-cell analyses of cells from adult mouse hearts sorted on the basis of surface markers identified cell-type specific transcripts that could be used to reveal the presences of multiple different cell types, including cardiomyocytes, fibroblasts, endothelial cells, marcophages, lymphocytes, granuloytes, smooth muscle cells, and Schawnn cells, in complex tissue samples^49,50^. Several of these cellular makers were identified in our human cardiac tissues, consistent with the presence of multiple cell types. Future RNASeq studies on at least some cell types, e.g., cardiomyocytes, isolated from different regions of the human heart will increase our understanding of the chamber-specific functional specialization of the human heart as revealed by transcriptomics.

## Conclusions

Comprehensive RNA sequencing revealed that the mRNA and lncRNA expression profiles in different regions of the non-failing human heart are distinct. Differential expression analysis of donor-paired samples identified regional differences in the expression levels of large numbers of mRNA and lncRNA transcripts in the atria and ventricles, as well as in the left and right sides of the heart. In addition, these results provide the first demonstration of regional differences in lncRNA expression in the human heart. Furthermore, we show here that the mRNA and lncRNA expression profiles in both atria and ventricles are distinct in males and females.

## Electronic supplementary material

Supplemental Material

Supplementary Dataset 1

Supplementary Dataset 2

Supplementary Dataset 3
