# Supplementary material for: Regional Differences in mRNA and lncRNA Expression Profiles in Non-Failing Human Atria and Ventricles
Source: Sci Rep. 2018 Sep 17;8:13919. doi: 10.1038/s41598-018-32154-2 (PMC6141608; doi:10.1038/s41598-018-32154-2)
Supplement: Supplementary file 1 — Supplemental Material [file 41598_2018_32154_MOESM1_ESM.pdf]

SUPPLEMENTAL MATERIAL

Regional Differences in mRNA and lncRNA Expression Profiles in Non-Failing Human Atria and Ventricles

Eric K. Johnson, PhD[1], Scot J. Matkovich PhD[1], and Jeanne M. Nerbonne, PhD[1,2]

[1]Department of Medicine, Cardiovascular Division, and
[2]Department of Developmental Biology,
Washington University School of Medicine, St. Louis, MO, 63110, USA

Supplemental Material

Supplemental Figure Legends

Supplemental Figure 1. Read count distributions of mRNA and lncRNA species in non-failing human LA, RA, LV, RV, and IVS are similar. (**A**) The vast majority (~86%) of total read counts in each region mapped to mRNAs, whereas the remaining (~14%) reads in each region mapped to lncRNAs. (**B**) The majority (~75%) of mRNA read counts reflect nuclear transcripts, whereas the remaining (~25%) of the total reads mapped the mitochondrial genome.

Supplemental Figure 2. **Distinct cellular components are enriched in non-failing human atrial and ventricular samples.** Maps of significantly (P < 0.05) enriched cellular components were generated using the Enrichment map tool[28] as described in **Methods and Materials**. Nodes represent individual cellular component terms from analyses of differentially expressed mRNA gene sets in the LA, compared with the LV (**A**), and in the RA, compared with the RV (**B**). Clusters of similarly enriched cellular components are indicated.

Supplemental Figure 3. **Total numbers of differentially expressed mRNA (A) and lncRNA (B) transcripts in non-failing human ventricles.** Differentially expressed mRNAs in the LV (black), RV (red) and IVS (grey) are indicated (all comparisons). The numbers of differentially expressed transcripts in the RV are higher than in the LV or IVS.

Supplemental Figure 4. Correlated expression of lncRNA-mRNA *cis* pairs identified in non-failing human atria and ventricles. Selected spearman's correlation analyses[16] of positive and negative correlations in the expression levels of several ion channel subunit transcripts (mRNAs) and lncRNAs identified in individual cardiac regions. Size factor-normalized read counts in for the lncRNA-mRNA *cis* pairs (N=8), in the specified region, are plotted and correlation coefficients (r) are indicated. [‡,§]Values indicated are significantly different at the $^{‡}P$ < 0.001 and $^{§}P$ < 0.0001 levels.

Supplemental Figure 5. Comparison of the non-failing human RNAseq data obtained in the present study with previously published non-failing human LV apex RNAseq data. Mean FPKM data from LA, LV, RA, RV and IVS samples obtained in this study were compared to mean LV transmural apex FPKM data from Yang et al., Circulation 2014 (NCBI GEO GSE46224; https://www.ncbi.nlm.nih.gov/geo/query/acc.cgi?acc=GSE46224). Scale for both axes is log2(FPKM+1). mRNAs for which mean FPKM = 0 in both data sets were not plotted. Linear correlations were calculated using Microsoft Excel.

Supplemental Figure 6. Comparison of the non-failing human RNAseq data obtained in the present study with previously published non-failing human heart RNAseq data. Mean FPKM data from LA, LV, RA, RV and IVS samples obtained in this study were compared to mean cardiac RPKM data from the Medicalgenomics RNAseq Atlas (references PMID 22345621 and PMID 20668672; http://medicalgenomics.org/rna_seq_atlas). Scale for both axes is log2(FPKM+1). mRNAs for which mean FPKM = 0 in both data sets were not plotted. Linear correlations were calculated using Microsoft Excel.

Supplemental Table 1. Summary of Donor Demographics[1]

Donor	Age	Sex	BMI	Ethnicity	Cause of death	EF
1	35	F	40	African American	Intracranial hemorrhage	N/A
2	65	F	21	Caucasian	Anoxia	N/A
3	54	M	28	Caucasian	Intracranial hemorrhage	75-80%
4	32	M	26	Caucasian	Motor Vehicle Accident	N/A
5	61	M	24	Caucasian	Cerebrovascular/stroke	60%
6	35	M	24	African American	Cerebrovascular/stroke	74%
7	47	M	25	Caucasian	Anoxia	60%
8	50	M	24	Hispanic	Cerebrovascular/stroke	60-65%

[1]BMI = Body Mass Index; EF = Ejection Fraction

Supplemental Table 2. Primers for Quantitative Real-time PCR

Gene	Forward Primer	Reverse Primer
XIST	3’ GCCCTACTAGCTCCTCGGACAG 5’	3’ CTAAGGACACATGCAGCGTGGTA 5’
EIF1AY	3’ TCTTCTGCGCACCCACCTG 5’	3’ CTGCGCCTGTTTTTACCTCCTT 5’
TRIPC1	3’ GCCCTCAAAGTGGTTGCTCAC 5’	3’ AGCCCTTCTGCCACCAGTGTA 5’
GABRA5	3’ CTTCCCTGCATAATGACCGTGAT 5’	3’ GAGTTCCTGGCGCTGATGCT 5’
SPN	3’ TCCCCCTCTTTCTTGTTCCTG 5’	3’ TTGCCGGGTTCTCTTGTCC 5’
DDN	3’ AGGCCCCGGGAACTCTCA 5’	3’ GTGCGCCCTCCGTCTGTC 5’
TBP	3’ CGGCTGTTTAACTTCGCTTCC 5’	3’ GGGGTCAGTCCAGTGCCATAA 5’

Supplemental Table 3. Summary of RNASeq Alignments[1]

	Read Pairs	% Aligned	Unique Read Pairs	Total mRNAs (≥1CPM)	Total lncRNAs (≥1CPM)
**Donor 1**					
LA	29,291,835	85.1%	27,050,101	13,247	6,310
RA	38,177,917	84.2%	38,135,410	13,057	8,954
LV	9,799,791	85.5%	22,019,436	12,649	4,899
RV	24,204,504	86.1%	24,547,282	13,231	5,502
IVS	30,725,614	84.4%	19,109,694	12,738	4,223
**Donor 2**					
LA	18,045,482	82.3%	24,154,368	12,358	7,374
RA	29,319,774	80.8%	31,129,012	12,372	8,672
LV	31,666,674	81.4%	8,121,701	12,585	2,558
RV	16,237,835	81.9%	20,268,307	12,682	5,688
IVS	15,125,037	83.1%	25,139,300	12,241	6,765
**Donor 3**					
LA	32,060,708	87.3%	14,368,156	13,053	4,016
RA	45,116,692	87.3%	22,858,794	13,397	6,922
LV	25,877,789	88.0%	24,938,759	12,548	6,705
RV	28,845,700	88.0%	12,826,247	12,727	4,170
IVS	22,533,826	87.7%	12,171,076	12,596	3,573
**Donor 4**					
LA	41,795,136	87.4%	35,440,620	13,537	8,974
RA	23,949,389	86.8%	20,122,165	13,685	6,273
LV	32,203,693	86.9%	27,263,221	12,917	6,340
RV	21,842,726	87.4%	18,587,542	13,149	5,015
IVS	26,256,647	87.8%	18,635,776	12,577	4,566
**Donor 5**					
LA	24,089,724	76.4%	17,745,702	13,370	6,267
RA	5,654,079	76.9%	4,190,947	13,442	1,822
LV	22,761,507	76.6%	16,839,458	12,682	5,099
RV	25,338,795	77.4%	18,971,172	12,843	6,033
IVS	12,928,297	77.2%	9,586,521	12,585	2,968
**Donor 6**					
LA	19,823,780	82.3%	15,712,549	13,148	4,666
RA	40,910,218	82.5%	32,581,766	13,198	8,547
LV	24,826,588	81.6%	19,532,654	12,632	4,983
RV	16,586,010	82.6%	13,212,974	12,780	3,834
IVS	26,256,647	82.5%	20,903,722	12,495	5,140
**Donor 7**					
LA	21,787,432	84.5%	17,740,947	13,391	5,645
RA	41,236,622	85.0%	33,778,530	13,417	9,284
LV	26,076,660	83.7%	21,030,542	12,719	5,284
RV	43,142,277	84.8%	35,232,203	12,771	7,205
IVS	14,227,609	85.0%	11,669,160	12,607	2,968
**Donor 8**					
LA	12,419,956	80.5%	9,582,616	12,923	3,071
RA	35,912,238	79.1%	27,452,696	13,227	7,514
LV	33,309,797	78.8%	25,348,611	12,600	6,210
RV	23,006,073	79.8%	17,730,082	12,706	4,512
IVS	15,682,169	80.2%	12,188,831	12,610	3,760

[1]CPM = Counts per million reads

Supplemental Table 4. Example Enriched Signaling Pathways in Non-Failing Human Ventricles[1]

KEGG Pathway	KEGG Accession #	Enriched Region	Fold Enrichment	*P* Value
**RV vs LV**				
Biosynthesis of unsaturated fatty acids	hsa01040	RV	13.5	2.0E-02
PPAR signaling	hsa03320	RV	10.8	3.8E-05
Tyrosine metabolism	hsa00350	RV	8.8	4.4E-02
Fatty acid metabolism	hsa01212	RV	8.6	1.1E-02
Glutathione metabolism	hsa00480	RV	8.1	1.3E-02
Steroid hormone biosynthesis	hsa00140	RV	7.1	1.8E-02
Glycolysis / Gluconeogenesis	hsa00010	RV	6.2	2.6E-02
cAMP signaling	hsa04024	LV	5.6	3.0E-02
AMPK signaling	hsa04152	RV	4.2	2.9E-02
Insulin signaling	hsa04910	RV	3.7	4.2E-02
**RV vs IVS**				
PPAR signaling	hsa03320	RV	4.6	3.9E-03
TNF signaling	hsa04668	RV	3.7	2.6E-03
Adipocytokine signaling	hsa04920	RV	3.8	2.1E-02
AMPK signaling	hsa04152	RV	3.6	1.7E-03
VEGF signaling	hsa04370	RV	3.6	4.8E-02
Insulin signaling	hsa04910	RV	3.2	3.9E-03
TGF-beta signaling	hsa04350	RV	3.1	4.1E-02
Insulin resistance	hsa04931	RV	2.9	3.5E-02
Carbon metabolism	hsa01200	RV	2.7	4.2E-02
MAPK signaling	hsa04010	RV	2.2	1.2E-02
GABAergic synapse	hsa04727	IVS	10.5	5.7E-03
**LV vs IVS**				
TNF signaling	hsa04668	LV	9.6	1.5E-03
Jak-STAT signaling	hsa04630	LV	5.6	3.1E-02
MAPK signaling	hsa04010	LV	4.0	3.2E-02
Serotonergic synapse	hsa04726	IVS	41.5	3.2E-02

[1]Example Kyoto Encyclopedia of Genes and Genomes (KEGG) pathways differentially represented in the paired LV (N=8), RV (N=8) and IVS (N=8) samples, determined as described in **Methods and Materials**.

Supplemental Table 5. Selected Differentially Expressed Ion Channel Subunits in Human Atria versus Ventricles[1]

		LA > LV		RA > RV	
**Gene**	**Protein**	**FD**	**FDR**	**FD**	**FDR**
**Atria**					
KCNA5	Kv1.5	22.7	4.1E-86	30.7	4.5E-49
KCNA6	Kv1.6	4.0	3.4E-15	2.5	1.1E-14
KCNJ3	Kir3.1	42.3	1.1E-52	33.4	1.6E-19
KCNJ5	Kir3.4	1.9	5.5E-08	2.2	5.1E-09
KCNK1	Kir1.1	5.9	5.5E-36	3.4	3.0E-26
KCNK3	K_{2p}3.1	27.0	1.4E-41	11.8	2.4E-28
KCNMB2	K_{Ca}4.1	4.7	1.3E-08	1.6	9.6E-04
KCNQ3	Kv7.3	4.0	4.4E-02	2.3	1.3E-04
SCN1B	Navβ1	3.0	1.1E-22	1.7	2.2E-05
CACNA1D	Cav1.3	11.1	8.1E-24	16.3	2.3E-43
CACNA1G	Cav3.1	43.0	1.7E-48	14.6	7.0E-38
CACNA2D2	Cavα2δ2	4.9	1.9E-38	19.8	1.9E-100
HCN1	HCN1	69.4	2.2E-146	65.9	2.9E-88
**Ventricles**		**LV > LA**		**RV > RA**	
KCNA4	Kv1.4	1.9	2.3E-05	3.6	2.5E-13
KCNAB2	Kvβ2	2.7	6.8E-16	1.7	2.2E-03
KCNJ2	Kir2.1	6.4	5.4E-37	4.2	1.4E-29
KCNJ8	Kir6.1	2.9	6.4E-32	2.0	8.0E-15
KCNK6	K_{2p}6.1	2.2	9.4E-05	2.2	4.1E-07
SCN2B	Navβ2	2.4	6.0E-18	2.0	3.1E-08
SCN4B	Navβ4	1.5	2.3E-08	2.4	1.7E-10
SCN7A	Nav2.1	1.8	9.4E-09	1.6	5.2E-04
CACNA2D1	Cavα2δ1	2.1	1.8E-12	2.1	1.1E-07

[1]Relative fold differences (FD) in mRNA transcript expression levels were determined from differential expression analysis using EdgeR, as described in **Materials and Methods**. Transcripts encoding ion channel subunits with higher expression in the atria or ventricles are provided. The relative fold differences (FD) in expression and the false discovery rate values (FDR) are also provided.

Supplemental Table 6. Selected Differentially Expressed Ion Channel Subunits Within Human Atria and Ventricles[1]

Gene	Protein	FD	FDR	Higher Expression
**Atria**				
**LA *vs* RA**				
KCNJ4	Kir2.3	1.8	1.2E-05	RA
KCNJ5	Kir3.4	1.6	6.9E-05	RA
KCNK13	K_{2p}13.1	2.5	1.1E-05	RA
KCNK17	K_{2p}17.1	3.9	7.1E-09	RA
HCN2	HCN2	1.7	1.3E-04	RA
HCN4	HCN4	3.9	2.6E-28	RA
CACNA1D	Cav1.3	2.1	2.2E-08	RA
CACNA2D2	Cavα2δ2	4.1	8.0E-54	RA
KCNA4	Kv1.4	1.9	3.6E-04	LA
KCNMB1	BKbeta2	2.7	1.8E-11	LA
KCNMB2	BKbeta2	2.3	7.1E-06	LA
KCNN2	K_{Ca}2.2	1.9	3.1E-14	LA
KCNQ3	Kv7.3	2.0	3.1E-14	LA
SCN3A	Nav1.3	2.4	3.1E-11	LA
SCN9A	Nav1.7	2.0	1.9E-05	LA
SCN3B	Navβ3	1.8	3.7E-04	LA
SCN4B	Navβ4	1.6	4.6E-07	LA
**Ventricles**				
**RV *vs* LV**				
KCNK1	K_{2p}1.1	1.7	4.6E-09	RV
KCNK3	K_{2p}3.1	1.7	4.4E-02	RV
CACNA1E	Cav2.3	4.6	4.2E-08	RV
KCNJ4	Kir2.3	2.4	3.0E-05	LV
HCN2	HCN2	2.8	3.0E-03	LV
**RV *vs* IVS**				
CACNA1E	Cav2.3	2.5	2.6E-05	RV
KCNJ4	Kir2.3	1.8	5.3E-04	IVS
HCN2	HCN2	2.7	1.3E-03	IVS
**LV *vs* IVS**				
KCNJ3	Kir3.1	1.8	2.4E-02	IVS

[1]Differences in mRNA transcript expression levels were determined from differential expression analysis using EdgeR as described in **Materials and Methods**. Selected transcripts encoding ion channel subunits expressed at higher levels in different regions of the atria or ventricles are provided. The relative fold differences (FD) in expression and the false discovery rate values (FDR) are also provided.

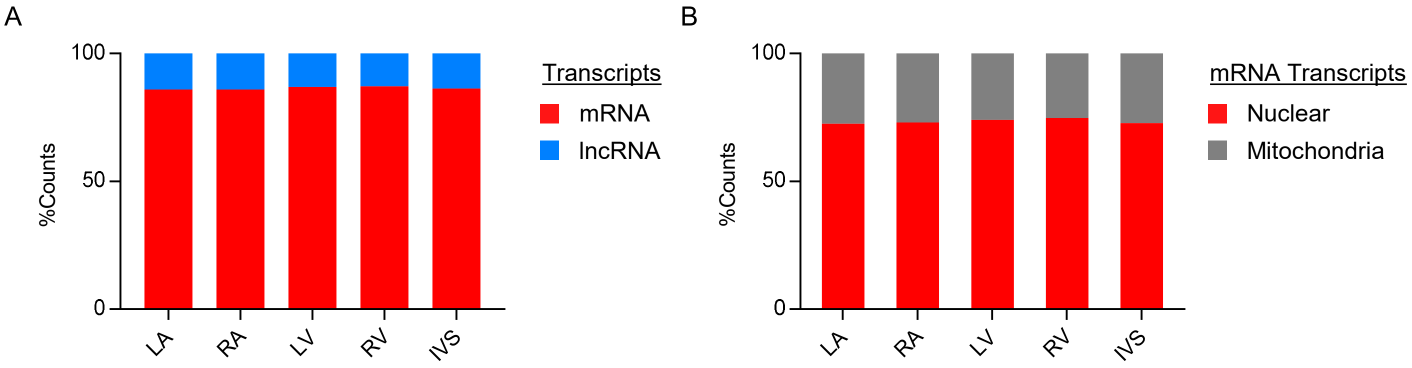

Supplemental Figure 1

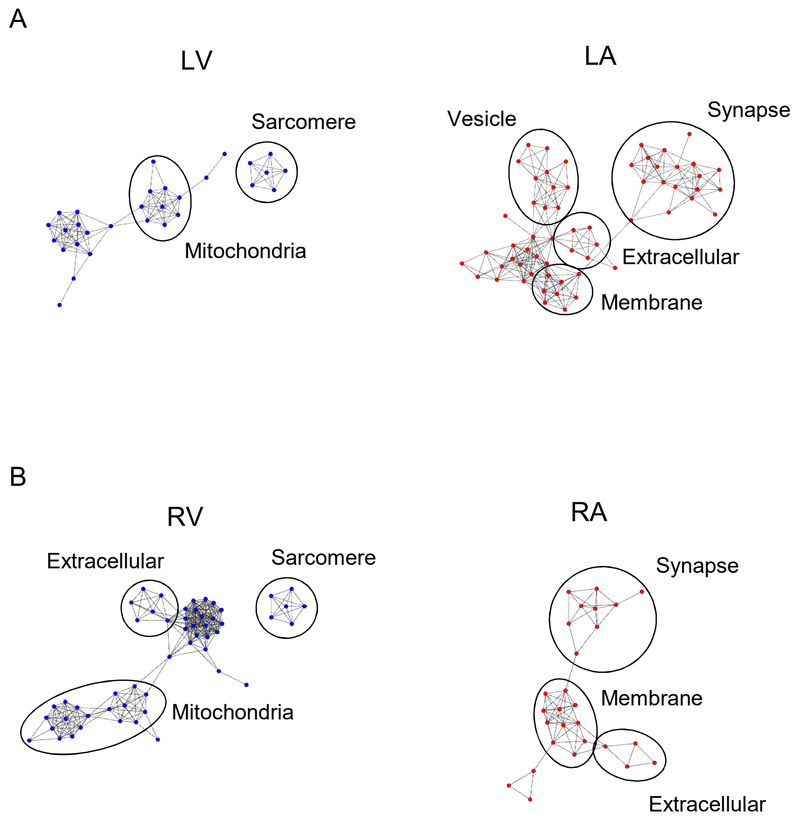

Supplemental Figure 2

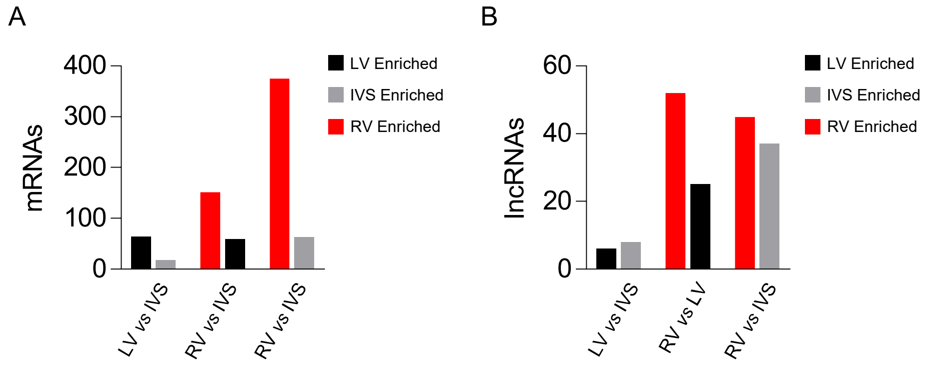

Supplemental Figure 3

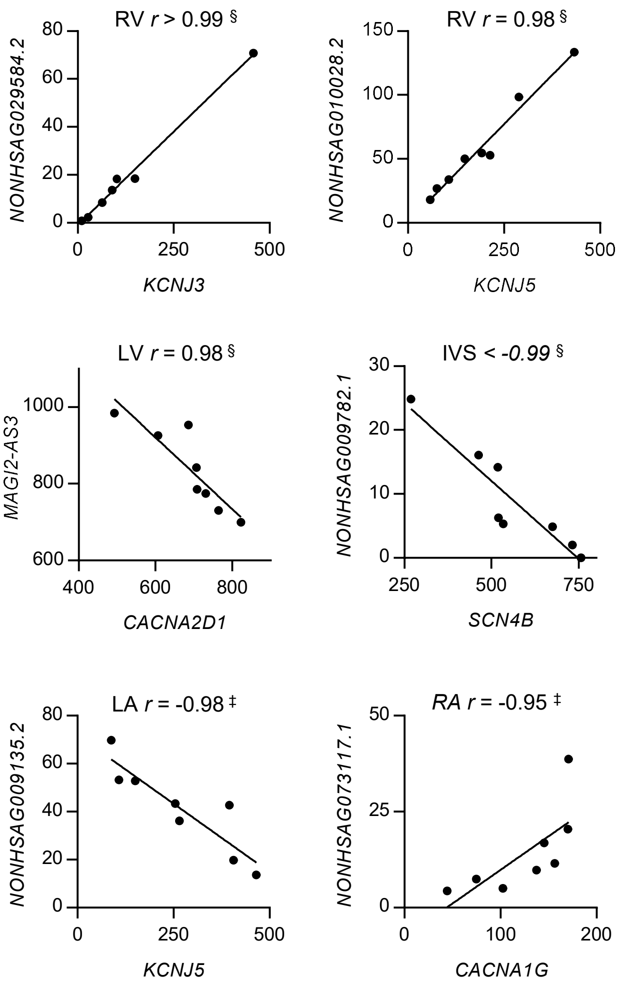

Supplemental Figure 4

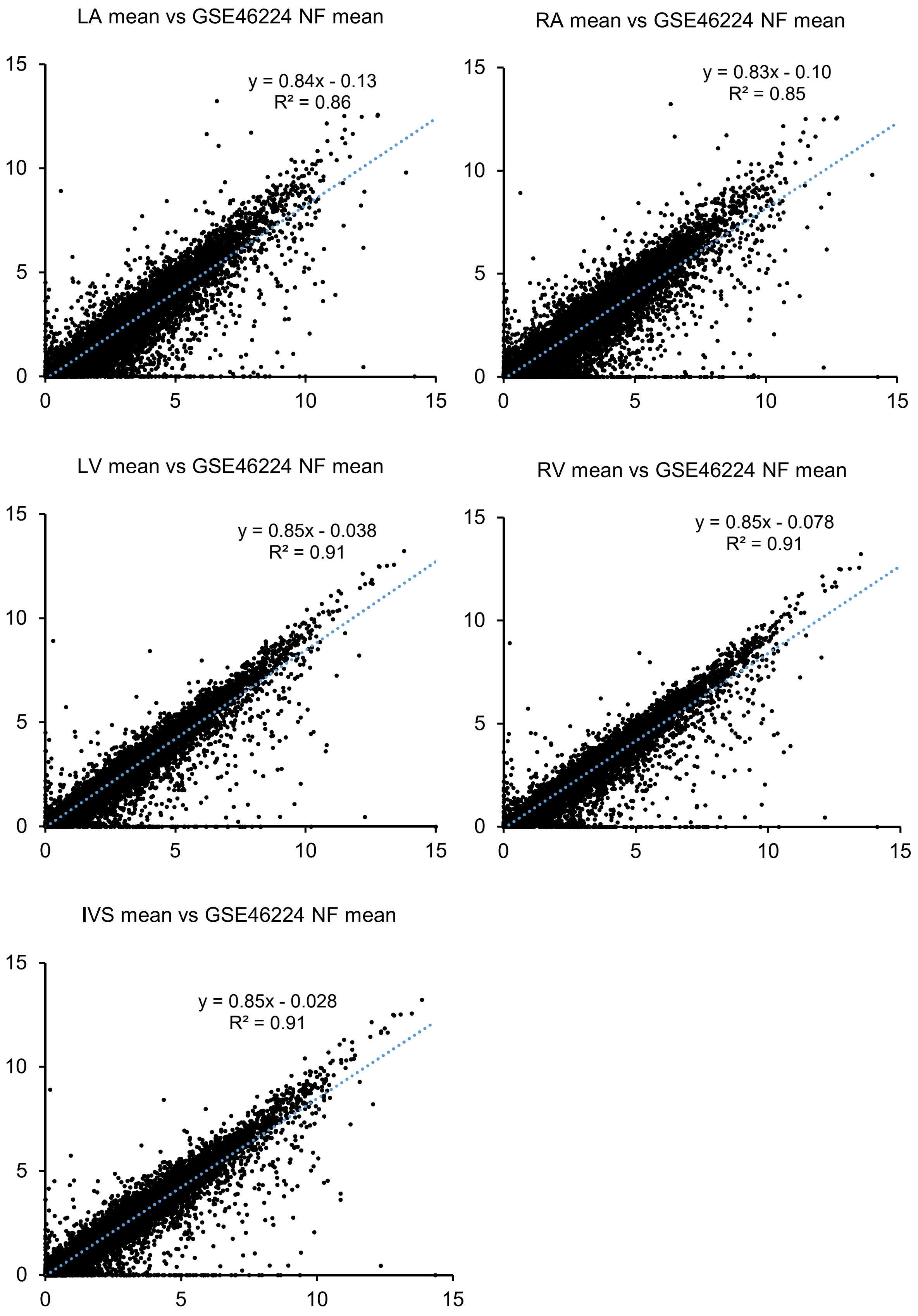

Supplemental Figure 5

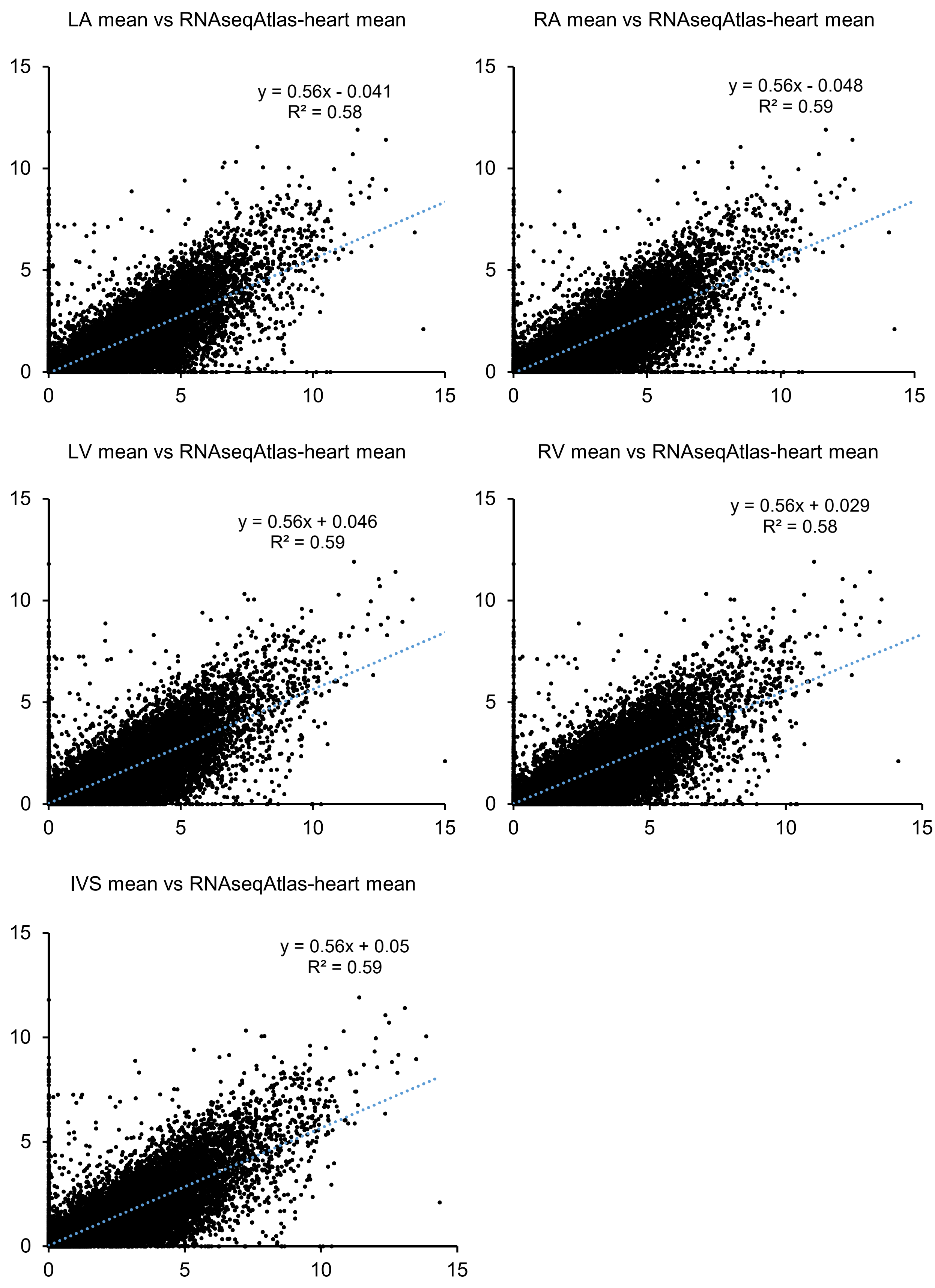

Supplemental Figure 6
